# The influence of placenta microbiota of normal term pregnant women on immune regulation during pregnancy

**DOI:** 10.1186/s12884-024-06353-x

**Published:** 2024-02-29

**Authors:** Ping Yang, Tong Lu, Xinyuan Liang, Ting Huang, Lulu Wu, Zonglin He, Xiaomin Xiao, Shangrong Fan

**Affiliations:** 1 Department of Obstetrics and Gynaecology, Peking University Shenzhen Hospital, Shenzhen Peking University-The Hong Kong University of Science and Technology Medical Center, Shenzhen, Guangdong Province China; 2 Department of Otolaryngology, Shenzhen Long Hua District Central Hospital, Shenzhen, China; 3 Shenzhen People’s Hospital, Shenzhen, China; 4 Department of Obstetrics and Gynecology, The First Affiliated Hospital of Jinan University, Guangzhou, China; 5 Division of Life Science, Hong Kong University of Science and Technology, Hong Kong, China

**Keywords:** Placental microbiota, Maternal immunity, Immune cells, Cytokines

## Abstract

**Background:**

The concerted regulation of placenta microbiota and the immune responses secures the occurrence and development of pregnancy, while few studies reported this correlation. This study aimed to explore the relationship between the placenta microbiota and immune regulation during pregnancy.

**Methods:**

Twenty-six healthy pregnant women scheduled for elective cesarean section in the First Affiliated Hospital of Jinan University who met the inclusion criteria were recruited. Placenta and peripheral venous blood samples were collected. Microbiota in placental tissue was detected using high-throughput sequencing. Flow cytometry was used to detect immune cells in placental tissue and peripheral venous blood. ELISA and Luminex liquid chip technology were used to detect the content of cytokines in placental tissue and peripheral venous blood, respectively.

**Results:**

The placental microbiota has stimulating effects on the local immunity of the placenta and mainly stimulates the placental balance ratio CD56 + CD16 + /CD56 + CD16 and the placental macrophages, that is, it plays the role of immune protection and supporting nutrition. The stimulating effect of placental microbiota on maternal systemic immunity mainly induces peripheral Treg cells and B lymphocytes.

**Conclusion:**

The placental microbiota may be an important factor mediating local immune regulation in the placenta, and placental microbiota participates in the regulatory function of the maternal immune system.

**Supplementary Information:**

The online version contains supplementary material available at 10.1186/s12884-024-06353-x.

## Background

The microbiota, consisting of trillions of microbes, resides within the mammalian intestinal tract, which is the body’s largest immune organ made up of cells that originate from both non-hemopoietic sources and hemopoietic sources such as macrophages, dendritic cells, and T-cells [1]. Studies have found that gut microbiota regulates the gut’s local and systemic immune function [2–7].

During pregnancy, the placenta, the organ for material exchange between the mother and the fetus, physiologically supports the growth and development of the fetus [8]. Much evidence shows that normal-term placental tissue contains much low-abundance microbiota [9–12], which may facilitate immune tolerance throughout pregnancy, from implantation and placentation to the growth and development of fetuses [13, 14]. The placenta is also a strictly regulated immune organ, which contains many immune cells such as Natural killer cells (NK cells), macrophages, T lymphocytes, and B lymphocytes [15–17]. Nevertheless, the interactions between the various microbiome in the placenta and maternal immunological responses remain elusive [18].

Aagaard et al. first reported the existence of the placental microbiome in healthy subjects and found that placental microbial composition has its own, unique composition [19], and there is tight correlation with the microbiome in the oral cavity, indicating that poor oral health may be associated with adverse pregnancy outcomes [20]. There are multiple hypothesis regarding the function of the microbial composition in the placenta. For instance, some researchers have postulated that the microbiome may act as the niche, where Ralstonia species [21], may exert symbiotic potentials to avoid inflammation and cell death while replicating in intracellular vacuoles of trophoblasts ex vivo [22, 23]. Secondly, bacterial cells have been found to be capable to be transferred via the placenta and play a role in early immune development, indicating their participation in the immunity aspect, which has not been clearly elucidated [24].

During physiological states, the most commonly found genus of microorganisms in the placenta is Lactobacillus, and interestingly, such species have been found to exist in both healthy gut and vaginal microbiome [25–27], as well as in human breast milk [28]. Following Lactobacillus, species like Ureaplasma, Streptococcus, Gemella, Veillonella, Prevotella, and Fusobacterium are also been widely reported to be existent in the placenta [29–31]. Moreover, some studies have reported that placental microbiota play important roles in adverse pregnancy outcomes, such as preterm birth and fetal macrosomia [11, 12, 32, 33]. Moreover, several researchers have reported that women with gestational diabetes mellitus had a higher abundance of Bacteroides, Bifidobacterium, Clostridium_XlVb, Duncaniella, Faecalibaculum, Harryflintia, Monoglobus, Ruminococcus, and Vampirovibrio in placenta samples [34, 35].

Therefore, based on the theory of gut microbiota’s mediation and stimulation of local gut immunity and the body’s systemic immunity, this study proposes that placental microbiota may play an important role in mediating placental local immunity and maternal immune stimulation and feedback.

## Materials and methods

### Design and study population

This study is a prospective cohort study. Twenty-six normal pregnant women who opted for elective Cesarean section in the First Affiliated Hospital of Jinan University and met the inclusion criteria were recruited, and informed consent was obtained. The inclusion criteria for this study were single pregnancy, Chinese nationality, having undergone routine prenatal check-up and delivery in the First Affiliated Hospital of Jinan University, natural conception, no complications during pregnancy and elective cesarean section at term (including those with a scarred uterus, incorrect fetal position, cephalopelvic disproportion and social factors). Exclusion includes gastrointestinal diseases, antibiotic use during pregnancy, history of hypertension and diabetes, hyperthyroidism, autoimmune diseases such as rheumatic diseases (such as rheumatic diseases), history of endocrine and metabolic diseases, and history of blood transfusion, organ transplantation or immunotherapy.

### Sample collection

Placental samples were collected under sterile conditions within 30 min of placental delivery upon surgery, to avoid the necrosis and focal calcification of the maternal surface of the placenta. We collected two placental tissues about 1 * 1 * 1 cm in size at a distance of 2 cm from the root of the umbilical cord with a depth of 1 cm. One of the fresh placental tissues was rinsed in phosphate buffer saline (PBS) solution to wash out the blood stains, and single-cell suspension and tissue homogenate was prepared subsequently. Placental immune cells and cytokines were detected by flow cytometry and ELISA, respectively. The rest of the placental tissue were then stored at—80° C for half an hour. 16S ribosomal DNA identification was conducted to detect placental microbiota.

Peripheral venous blood samples were collected before the full-term elective surgery, where we collected 2 ml of maternal peripheral venous blood and placed it in heparin-rinsed collection tubes. The blood samples were centrifuged to obtain a mononuclear cell suspension and plasma. The peripheral immune cells and cytokines were then detected by flow cytometry and Luminex liquid chip technology, and the rest was stored in a refrigerator at—80 ℃ for future use.

### Research method

#### Flow cytometry

Flow cytometry was performed to identify immune cells in the peripheral blood and placenta of the pregnant women. Specifically, the fresh placental tissue was rinsed with PBS and dried with filter paper before being cut into pieces and digested with 0.05% collagenase II and 0.01% Dnase I. Once the tissue became soft, it was agitated with a Babbitt straw and filtered through a 70um cell sieve to create a mononuclear cell suspension. Peripheral blood samples were collected, and the cloud-like cell layer was extracted by centrifugation, washed twice with PBS, and resuspended to create a mononuclear cell suspension. T lymphocyte subtypes (CD3 + CD4 + T, CD3 + CD8 + T, CD3 + CD4 + CD8 + T, CD4 + CD25 + Foxp3 (Treg cells)), B lymphocytes (CD3-CD19 + /CD3-), macrophages (CD3-CD68 + /CD3-), and NK cells (CD3-CD56 + /CD3-) and their subtypes (CD56 + CD16 + , CD56 + CD16-, CD56dimCD16 + , CD56highCD16 + , CD56highCD16-, CD56dimCD16-) were detected using 100ul of single cell suspension. The remaining cells were stored at -80 °C for future use.

#### Luminex liquid chip technology

Luminex liquid chip technology was utilized to detect cytokines in peripheral blood. This technology is based on microsphere detection and enables the analysis of multiple target molecules and cytokines in a single experiment. To elaborate, 1 ml of collected peripheral blood was first centrifuged for 10 min to obtain plasma, which was then stored at -80 °C for future use. Upon analysis 50ul of collected peripheral blood was used for detection of IL-1, IL-2, IL-4, IL-5, IL-6, IL-12, IL-13, IL-18, IFN-γ, TNF-a, and GM-CSF content.

### ELISA

ELISA was used to detect the contents of cytokines in placental samples. To elaborate, fresh placental tissue samples were first rinsed with PBS to remove blood, and then cut into pieces after being dried with filter paper. Next, 250ul of protein lysate was added per 50 mg of tissue, and the lysate was added to the tissue on ice and thoroughly homogenized. The mixture was then centrifuged for 5 min. The resulting supernatant was collected and stored at -80 °C. The stored samples were then analyzed using ELISA to determine the content of IL-1, IL-2, IL-4, IL-5, IL-6, IL-12, IL-13, IL-18, IFN-γ, TNF-α, and GM-CSF.

### Statistical analysis

Data was analyzed SPSS.23 and Cytoscape_ 3.6. The Spearman analysis method was used to analyze the correlation between placental microbiota, immune cells, and cytokines. Data is presented as mean ± SD. The result was statistically significant with a *p*-value < 0.05.

## Result

In this study, 26 pregnant women with elective cesarean section were recruited including 19 with scarred uterus and 7 with breech presentation, who had no intention of trial delivery, bloody show, and abdominal pain before the operation. The clinical data of the mother and newborn are as follows: age 32.19 ± 4.13 years, height 158.6 ± 4.49 cm, weight 69.12 ± 8.03 kg, BMI 27.52 ± 3.04 kg/m^2^, gestational age at delivery 275.77 ± 3.63 days, birth weight 3.24 ± 0.33 kg, length 48.92 ± 1.33 cm, head circumference 33.69 ± 1.19 cm, and Apgar score 9–10-10.

### Complexity analysis of placenta samples

**Fig. 1:**
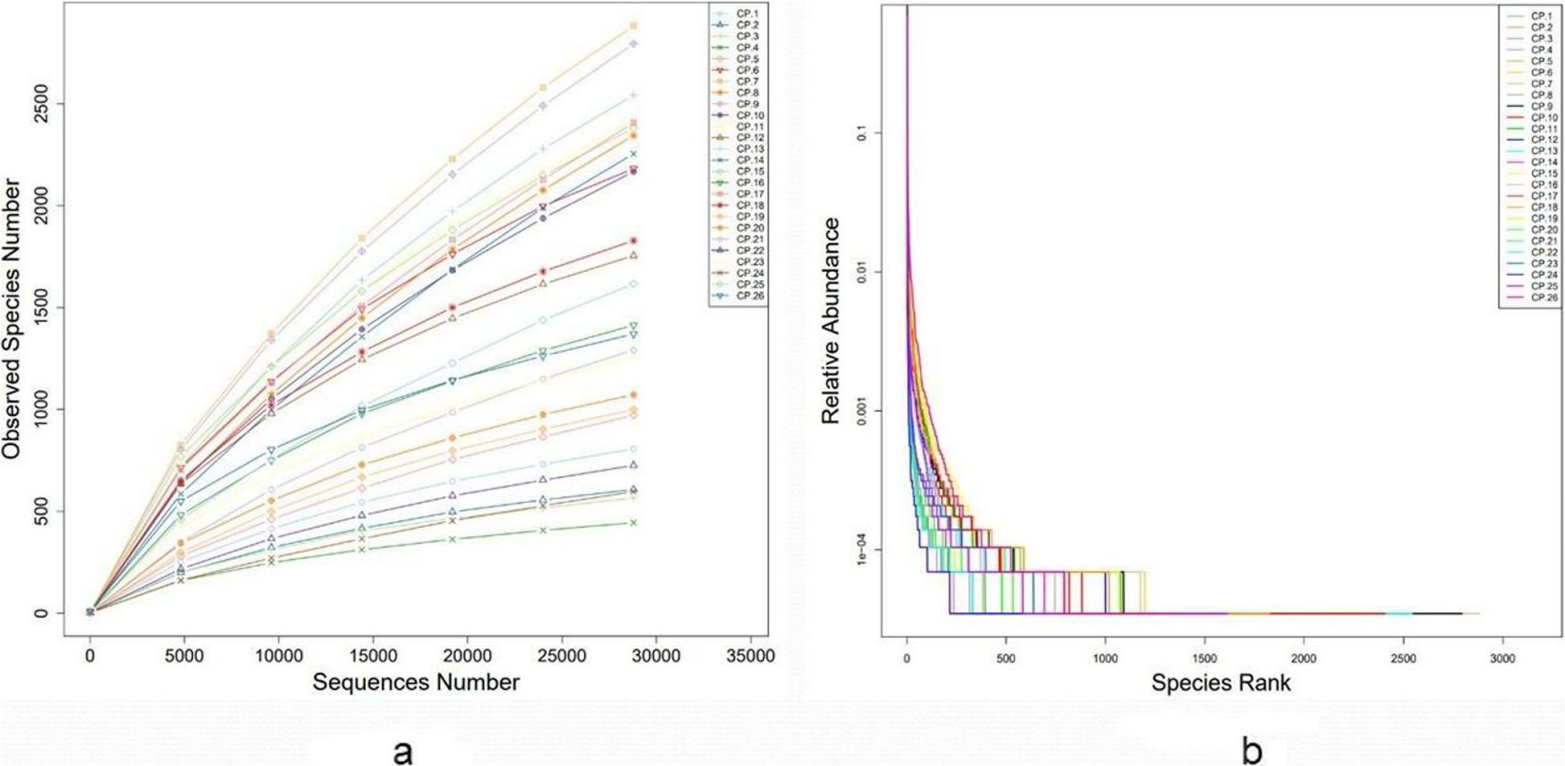
Dilution curve and rank abundance curve of placental microbiota of each sample

Through the dilution curve and Rank Abundance curve (Fig. 1a, b) of the placental microbiota (CP group) of each sample, it was found that the sample size of the placental tissue of normal full-term and elective cesarean section pregnant women in this study was sufficient. The data volume was reasonable, and the species in each placental microbiota were rich and evenly distributed.

**Fig. 2:**
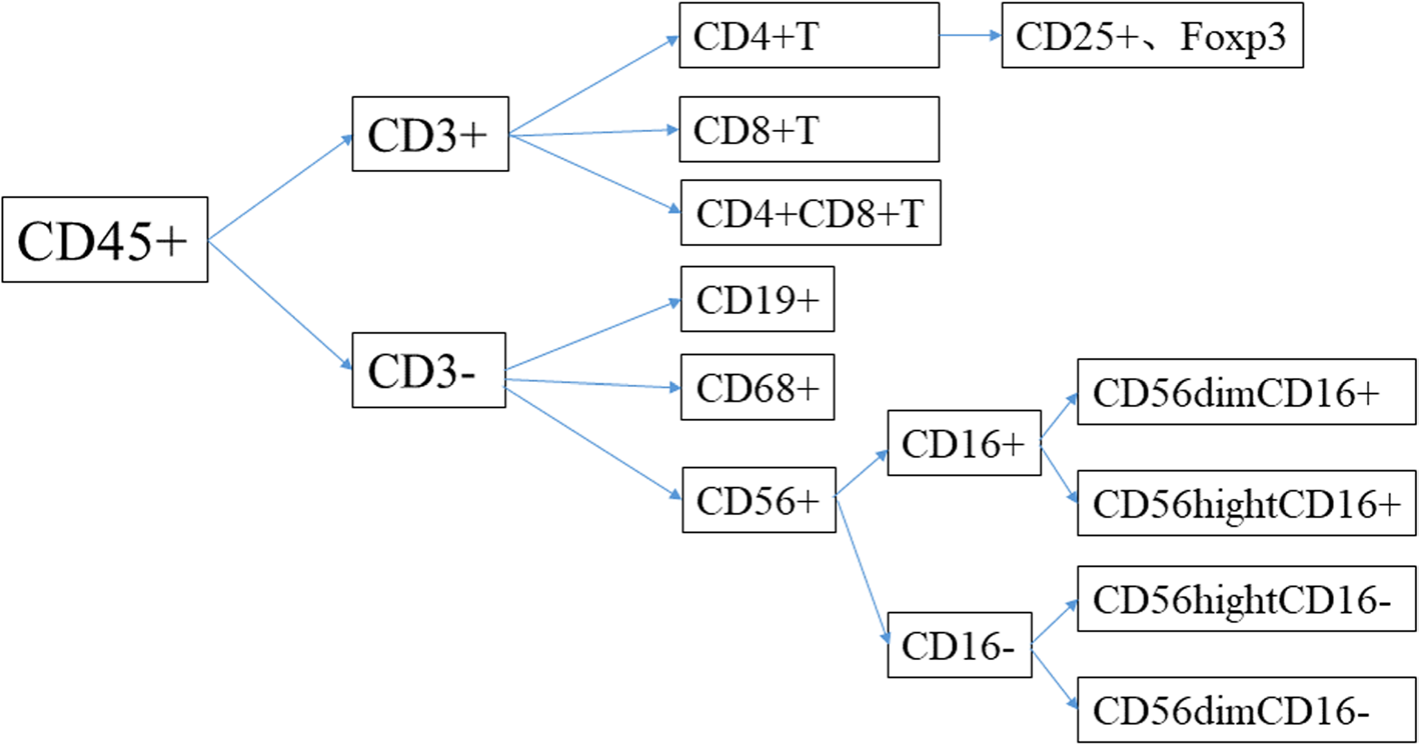
Immunocyte types

According to the distinct surface markers of lymphocytes, we used flow cytometry to classify the immune cells in peripheral blood and the placenta. Then, we calculate the percentage of T lymphocyte subtypes (CD3 + CD4 + T, CD3 + CD8 + T, CD3 + CD4 + CD8 + T, CD4 + CD25 + Foxp3 (Treg cells)), B lymphocytes (CD3-CD19 + /CD3-), macrophages (CD3-CD68 + /CD3-), and NK cells (CD3-CD56 + /CD3-) and their subtypes (CD56 + CD16 + , CD56 + CD16-, CD56dimCD16 + , CD56highCD16 + , CD56highCD16-, CD56dimCD16 -).The specific classification and subtypes of immune cells are shown in Fig. 2.

### Comparison of immune cells in maternal peripheral blood and placenta

Through the statistical test on the percentage and proportion of the above immune cells and their subtypes, this data conforms to the non-normal distribution, so the non-parametric rank sum test is used for the median comparison. The median and interquartile intervals (25th and 75th percentiles) were used to describe the entire data, and the Z and *P* values of the test statistics were calculated.

In Table 1, the percentage content of CD3 + CD4 + T, CD3 + CD8 + T, and the ratio of CD3-CD19 + /CD3—in peripheral blood were significantly higher than those in placental tissue, with statistically significant differences (all *P* < 0.05); The percentage contents of Treg cells (CD3 + CD4 + CD25 + FOXP3) and CD56dimCD16 + NK in placental tissue were significantly higher than those in peripheral blood (*P* < 0.05). The other immune cells and subtypes had no significant difference in peripheral blood and placental tissues (*P* > 0.05).

**Table 1:** Comparison of immune cells and their subtypes in peripheral blood and placental tissue

Immune cell index	Peripheral Blood M (P25, P75)	Placental Tissue M (P25, P75)	Z	*P*
CD3 + CD4 + T	43.35% (35.10%, 50.78%)	26.35% (16.85%, 36.68%)	2.609	0.009^*^
CD3 + CD8 + T	34.00% (27.48%, 39.05%)	22.80% (17.63%, 27.50%)	3.774	0.000^*^
CD4 + /CD8 +	1.16 (0.98, 1.73)	1.15 (0.82, 1.55)	0.175	0.861
CD3 + CD4 + CD8 + T	1.30% (0.60%, 6.45%)	1.40% (0.95%, 3.83%)	0.206	0.836
CD3 + CD4 + CD25 + FOXP3	0.60% (0.20%, 1.18%)	2.65% (1.73%, 4.15%)	3.944	0.000^*^
CD3-CD19 + /CD3-	0.46 (0.31, 0.55)	0.23 (0.13, 0.31)	3.900	0.000^*^
CD3-CD68 + /CD3-	0.02 (0.01, 0.11)	0.05 (0.03, 0.11)	1.658	0.097
CD3-CD56 + /CD3-	0.36 (0.25, 0.53)	0.29 (0.17, 0.49)	1.254	0.210
CD56 + CD16 + NK	7.15% (4.85%, 10.38%)	9.40% (4.73%, 15.00%)	1.037	0.300
CD56dimCD16 + NK	1.80% (1.18%, 3.68%)	3.70% (3.05%, 7.55%)	3.148	0.002^*^
CD56hightCD16 + NK	3.75% (2.30%, 8.88%)	3.15% (1.53%, 9.90%)	0.660	0.509
CD56 + CD16-NK	5.25% (3.25%, 9.28%)	6.30% (2.98%, 17.50%)	0.490	0.624
CD56hightCD16-NK	2.05% (1.30%, 2.53%)	1.70% (0.55%, 6.30%)	0.397	0.692
CD56dimCD16-NK	3.35% (1.98%, 6.95%)	3.70% (1.65%, 6.63%)	0.038	0.970
CD56 + CD16 + /CD56 + CD16-	1.50 (0.550, 2.48)	1.09 (0.58, 2.92)	0.000	1.000

^*^represents *P* < 0.05, with statistical significance

### Correlation between placental microbiota and placental immune cells

#### Network correlation diagrams

**Fig. 3:**
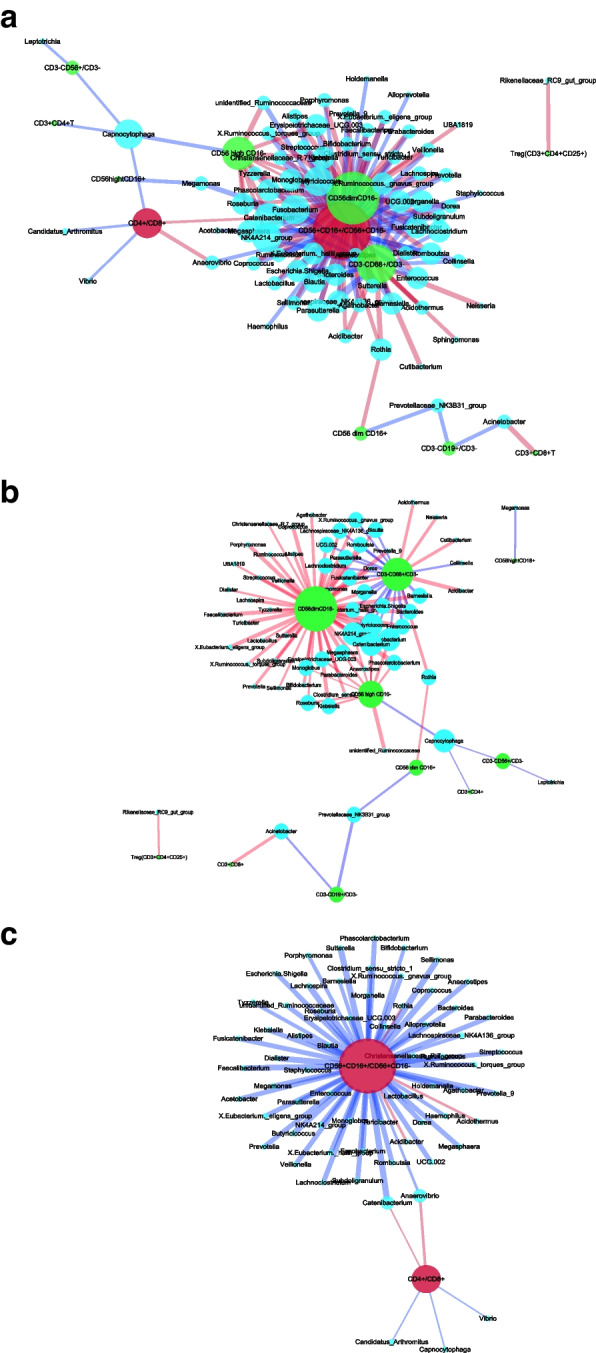
**a** Network correlation diagram between the top 100 bacteria of placenta microbiota and placental immune cells and balance state (overall diagram). **b** Network correlation diagram between the top 100 bacteria of placenta microbiota and placental immune cells (split diagram) (*P* < 0.05). **c** Network correlation diagram between the top 100 bacteria of placental microbiota and placental immune balance (split diagram) (*P* < 0.05)

Blue dots represent the genus, green dots represent immune cells, purple dots represent cytokines, red dots represent equilibrium ratios, blue lines represent negative correlations, red lines represent positive correlations, thickness represents the size of correlation coefficients, and the size of dots to indicate the strength/weakness of correlation. The Spearman correlation analysis was carried out between the top 100 bacterial genera of normal-term placental microbiota and immune cells in placenta tissue, and network correlation diagrams were obtained, as shown in Fig. 3a. The number of placental CD3 + CD4 + T, CD3 + CD8 + T, CD3 + CD4 + CD8 + T, CD3 + CD4 + CD25 + FOXP3, and CD3-CD19 + /CD3- significantly correlated with placental microbiota is very small (Fig. 3b). Placental macrophages (CD3-CD68 + /CD3 -) were positively correlated with 6 genera and negatively correlated with 19 other genera (*P* < 0.05) (Fig. 3b). There is a complex network relationship between placental microbiota and immune cells. The regulation of immune cells is mainly to maintain the immune balance of the body. Therefore, in this study, we chose two ratios of CD56 + CD16 + /CD56 + CD16—and CD4 + /CD8 + to represent the balance of placental immune cells. As shown in Fig. 3c, the ratio of placental CD56 + CD16 + /CD56 + CD16—was negatively correlated with 59 bacterial genera in the placental microbiota, and only positively correlated with three bacterial genera, namely, Acidibacter, Acidothermus and Rothia (*P* < 0.05). However, the ratio of CD4 + /CD8 + in the placenta was significantly correlated with only five bacterial genera (*P* < 0.05). Placental microbiota mainly affects placental macrophages and CD56dimCD16- cells, which plays a nutritional protection role, and inhibits the phagocytosis and killing effect of macrophages; it reduced the ratio of CD56 + CD16 + /CD56 + CD16 -, but had less effect on the ratio of CD4 + /CD8 + .

### Correlation between placental microbiota and placental cytokines

**Fig. 4:**
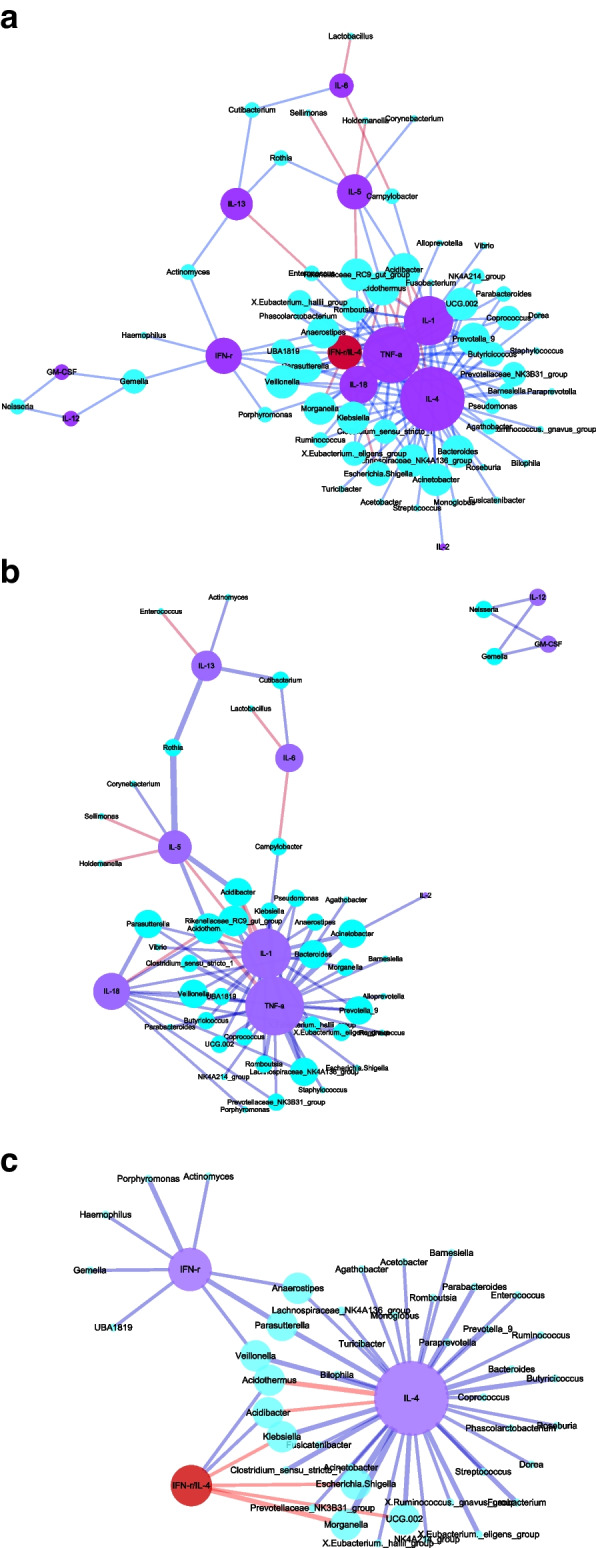
**a** Network correlation diagram between the top 100 microbiota of placenta and various cytokines and balance status of placenta (overall diagram) (*P* < 0.05). **b** Network correlation diagram between the top 100 bacteria of placenta microbiota and various cytokines of placenta (split diagram) (*P* < 0.05). **c** Network correlation diagram between the top 100 microbiota of placenta and the balance of placental cytokines (split diagram) (*P* < 0.05)

Since the activation and secretion of cytokines regulate the functions of immune cells, we conducted the Spearman correlation analysis between the cytokines in placenta tissue and the top 100 bacteria of placenta microbiota, and the network correlation diagrams were obtained, as shown in Fig. 4a. Specifically, only statistically significant correlations are shown.

As shown in Fig. 4b, the number of significant correlations between placental cytokines IL-1, TNF-a, IL-18, and placental microbiota was 22, 30, and 10, respectively, and they were all negatively correlated, indicating an antagonizing relationship. However, the number of significant correlations of placental IL-2, IL-5, IL-6, IL-12, IL-13, and GM-CSF significantly correlated with placental microbiota was minimal (Fig. 4b). As indicated in Fig. 4c, there was a significant negative correlation between factor IFN- γ and 8 kinds of bacteria in the placenta microbiota. The number of significant correlations between IL-4 and placental microbiota was up to 38, and 36 of them were negative correlations, and a positive correlation was only found between IL-4 and Acidothermus and Acidibacter. Placental microbiota had the strongest regulatory effect on IL-4; the inhibitory effect was dominant. There are 6 bacterial genera with a significant correlation between the factor IFN-γ/IL-4 and placental microbiota, among which there is significant positive correlation between the factor and two bacterial genera, namely, Acidothermus and Acidibacter. Therefore, the correlation between placental microbiota and factor IL-4 is the greatest in placental tissue and they are is negatively correlated.

### Correlation between placental microbiota and maternal systemic immunity

**Fig. 5:**
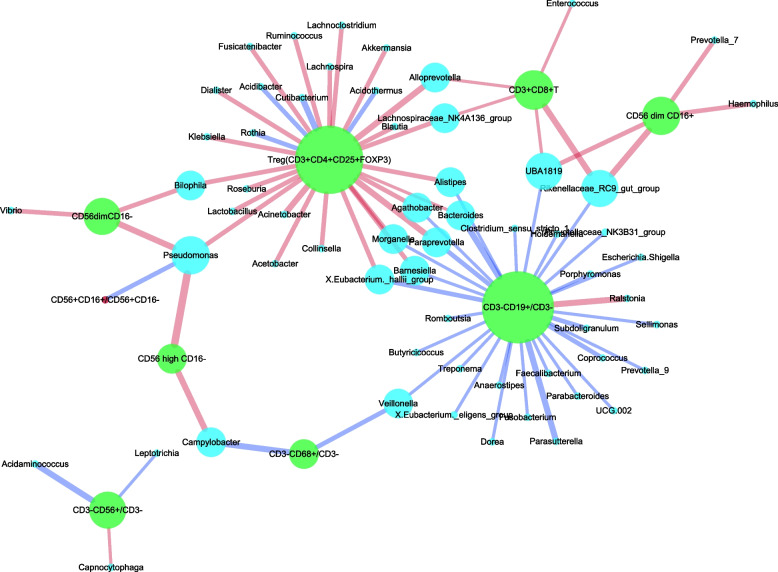
Network correlation diagram between the top 100 bacteria of placenta microbiota and peripheral immune cells and balance state (*P* < 0.05)

Later, the Spearman correlation analysis was conducted to indicate the relationship between the top 100 genera of placental microbiota and their T lymphocyte subtypes, B lymphocytes, macrophages and NK cell subtypes in the peripheral blood, and the network correlation diagrams were obtained, as shown in Fig. 5. Similarly, only statistically significant correlations were visualized.

### Correlation between placental microbiota and peripheral blood immune cells

The number of peripheral Treg cells significantly correlated with placental microbiota was 28, and the positive correlation was dominant (i.e., mutual promotion).

CD3 + CD4 + T and CD3 + CD4 + CD8 + T had no significant correlation with placental microbiota (*P* > 0.05). The number of peripheral B lymphocytes with a significant correlation with placental microbiota was 31 at most, and the negative correlation was dominant (mutual inhibition); that is, the influence of peripheral B lymphocytes was the strongest. However, the number of peripheral macrophages and NK cell subtypes significantly correlated with placental microbiota was less. There is a complex network relationship between the placental microbiota and the peripheral immune cells, and the regulation of the immune cells is mainly to maintain the immune balance of the body. The ratio of peripheral CD56 + CD16 + /CD56 + CD16—had a significant negative correlation with only one genus, the Pseudomonas, while the ratio of peripheral CD4 + /CD8 + had no significant correlation with placental microbiota (*P* > 0.05). Therefore, the negative regulation of main peripheral B lymphocytes and the positive regulation of peripheral Treg cells on the placental microbiota are dominant in the peripheral blood. In contrast, the regulation of peripheral macrophages, NK cell subtypes, and their balance ratios on the placental microbiota is minor.

### Correlation between placental microbiota and peripheral blood cytokines

Through Luminex liquid chip technology, we detected the levels of cytokines in the peripheral blood, namely, IL-1, IL-4, IL-6, IL-13, IL-18, TNF-a, IFN- γ and IFN- γ/IL-4. The Spearman correlation analysis was conducted between the peripheral cytokines and the top 100 strains of placental microbiota, and network correlation diagrams were obtained.

**Fig. 6:**
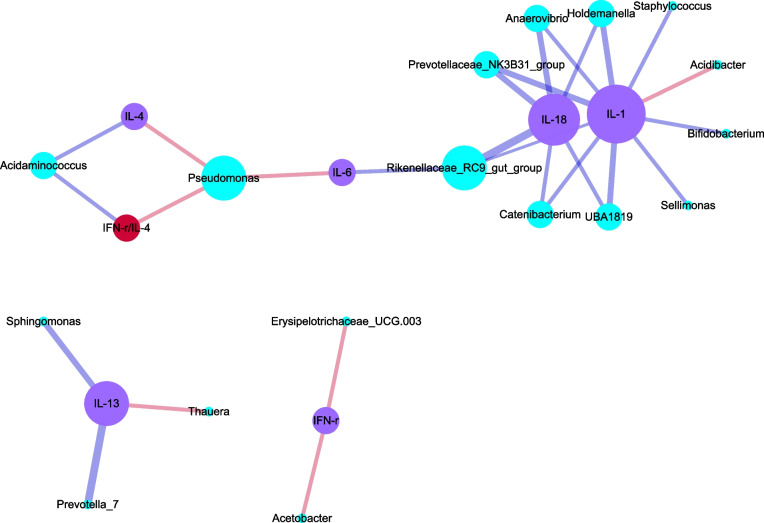
Network correlation diagram between the top 100 bacteria of placenta microbiota and peripheral cytokines and balance state (*P* < 0.05)

As shown in Fig. 6, the number of significant correlations of either IFN-γ or IL-4 with placental microbiota was 2. Peripheral IL-1 was found to have up to 10 significant correlations with the placental microflora, with most of them being negative. There was a significant negative correlation between peripheral IL-18 and placental microbiota, whereas no significant correlation was found between peripheral TNF-a and placental microbiota. Only a few significant correlations were observed between IL-6 and IL-13 and placental microbiota. The ratio of peripheral IFN-γ/IL-4 was significantly correlated with two genera of placental microbiota but had a lesser effect than peripheral cytokine IL-1. In conclusion, the strongest correlation was observed to be between peripheral cytokine IL-1 and the placental microbiota, and the correlation was negative. On the other hand, the peripheral IFN-γ/IL-4 ratio had a relatively weaker correlation with the placental microbiota.

## Discussion

### Effect of placental microbiota on local placental immunity

Studies have shown that many microbiota existing in mammals have a highly coevolutionary relationship with the immune system [36, 37]. The immune system of mammals plays a crucial role in maintaining the balance of the internal environment with the resident microbial community, to ensure that the reciprocity of the host microbiota relationship is maintained. At the same time, the resident microbial community profoundly affects the immunity of mammals. As we all know, the maintenance of normal and successful pregnancy cannot be separated from the regulation of the local immune system of the placenta [38]. The placenta is a strictly regulated immune organ, containing many immune cells, such as NK cells, macrophages, T cells, and trophoblasts with immune regulation functions. These immune cells participate in the formation of the maternal–fetal interface and play an important role in maintaining normal pregnancy and maternal–fetal immune tolerance [39]. We have previously confirmed that the placenta of normal-term pregnant women contains microorganisms, and there are many species, mainly low-abundance bacteria. Its microbiota composition significantly differs from the mother’s intestinal and oral microbiota. Alpha diversity is also significantly higher than that of the mother’s intestinal and oral microbiota, and a complex network relationship is found between the microbiota. Some scholars found that these symbionts mainly existed in the extravillous trophoblasts at the maternal–fetal interface through morphological studies [9]. So, do trophoblasts expressing pattern recognition receptors receive the stimulation of placental microorganisms to start regulating maternal–fetal interface immune function? At present, there is no relevant report.

The present study found that the placental microbiota may influence the placental immune cells CD56dimCD16 -. Therefore, this may indirectly affect the function of the placenta and the growth and development of the fetus, and the imbalance between them will lead to pathological pregnancy. The placental microbiota can inhibit the phagocytosis and killing effect of placental macrophages, reduce the ratio of placental immune balance CD56 + CD16 + /CD56 + CD16-. This may be beneficial for maintaining a normal pregnancy and ensuring fetal health. However, the effect between placental microbiota and placental immune balance CD4 + /CD8 + is less. At the same time, there is a positive correlation between Acidobacter and Acidothermus and the CD56 + CD16 + /CD56 + CD16—ratio of the placenta, which can increase the CD56 + CD16 + /CD56 + CD16—ratio of the placenta, thus maintaining the homeostasis of placental immune cells.

Because each immune cell produces immunization by secreting different cytokines and acting on the corresponding target cells to play an immune regulatory role. It can be seen from the network correlation diagram between the first 100 bacteria and placental cytokines in the placental microbiota that placental cytokine TNF-a is negatively correlated with 29 bacteria of the placental microbiota, that is, placental microbiota can inhibit the content of placental TNF-a (Fig. 4b, c). TNF-a is a proinflammatory cytokine, which is mainly secreted by macrophages, and also acts on macrophages. This study concluded that placental microbiota may inhibit the role of placental macrophages (phagocytosis and killing). Moreover, higher levels of TNF-a might lead to a lower frequency of placental bacteria due to its cytotoxicity, thereby potentially explaining the observed negative correlation. So it is speculated that placental microbiota may inhibit the role of macrophages by inhibiting the content of placental TNF-a, leading to the weakening of its target cells (macrophages). The number of significant correlations between placental cytokine IL-1 and placental microbiota was more than 22, and mainly negative correlation. IL-1 is an proinflammatory cytokine, mainly derived from macrophages, B lymphocytes and dendritic cells, and its corresponding target cells are B lymphocytes, NK cells and T lymphocytes. Therefore, placental microbiota reduces the secretion of placental cytokine IL-1 by inhibiting the proliferation of macrophages. The number of significant correlations between placental IL-4 and placental microbiota was 38 at most, and 36 of them were a negative correlation, only positive correlation was found between placental IL-4 and Acidothermus and Acidibacter, that is, placental microbiota had the strongest regulatory effect on placental IL-4, and the inhibitory effect was dominant. IL-4 is a Th2 cytokine; its target cells are B lymphocytes, T lymphocytes and macrophages. Therefore, placental microbiota can also inhibit the phagocytosis of macrophages by inhibiting the content of IL-4.

In conclusion, placental microbiota can comprehensively inhibit the content of placental cytokines IL-1, TNF-a and IL-4, thereby inhibiting the phagocytosis of placental macrophages. This study shows a negative correlation between placental microbiota and placental IFN- γ, that is, placental microbiota will inhibit the content of cytokine IFN- γ. Factor IFN- γ as an important cytokine, it plays an important role in immune regulation in the human body, mainly produced by NK cells and activated T lymphocytes. It can enhance the effect of CD3 + CD8 + T and NK cells (i.e. cytotoxicity). Factor IFN-γ, that is, a type of Th1 cytokine. If it exists in large quantities, it will lead to an imbalance between cytokines, which will be detrimental to maintaining normal pregnancy. The increase in its number can lead to excessive inflammatory reaction in the maternal systemic circulation. It is speculated that normal term placental microbiota can inhibit placental IFN- γ and reduce NK cell activation, thereby reducing the ratio of CD56 + CD16 + /CD56 + CD16—in the placenta, which plays a nutritional and protective role in the fetus.

To sum up, the placental microbiota of normal term pregnant women interacts with placental local immunity, especially the influence of placental macrophages, CD56dimCD16-NK cells and placental microbiota; The placental microbiota has the most significant influence on the placental immune balance CD56 + CD16 + /CD56 + CD16—ratio, that is, it causes the ratio to decrease. Placental microbiota can comprehensively inhibit the content of placental cytokines IL-1, TNF-a and IL-4, thereby inhibiting the phagocytosis of placental macrophages. At the same time, placental microbiota inhibits placental factor IFN- γ and reduces the activation of NK cells, thereby reducing the ratio of placental balance CD56 + CD16 + /CD56 + CD16 -, which plays a nutritional and protective role in the fetus.

### Effect of placental microbiota on maternal systemic immunity

Microbes and the immune system of the body are complementary and interactional. The above research shows that the placental microbiota of normal term pregnant women interacts with the immune cells and cytokines in the placenta, especially the influence between the placental microbiota and the local immunity of the placenta. The regulating function of maternal system immunity runs through the whole body, so it is speculated that the placental microbiota and immune cells in the peripheral blood also interact. Therefore, this study conducted the Spearman correlation analysis between the top 100 bacteria in the placenta microbiota and the immune cells in the peripheral blood. As you can see in Fig. 5, the negative regulation of main peripheral B lymphocytes and the positive regulation of peripheral Treg cells on placental microbiota are dominant in peripheral blood. In contrast, the regulation of peripheral macrophages, NK cell subtypes and their balance ratio on placental microbiota is minor. This suggests that the influence of B lymphocytes on peripheral blood and placental microbiota is greater than that on placental local B lymphocytes and placental microbiota. Although the percentage content of Treg cells in the peripheral blood is small, it has a greater impact on the placental microbiota, and mainly increases the relative abundance of bacteria, thus indirectly reducing the peripheral immune balance ratio CD56 + CD16 + /CD56 + CD16 -, and enhancing the immune protection and nutrition of the fetus.

The influence between peripheral cytokine IL-1 and placental microbiota is the greatest, and the inhibition is the main effect. Because IL-1 can be secreted by B lymphocytes and acts on B lymphocytes and T lymphocytes, the influence between peripheral B lymphocytes and Treg cells and placental flora is greater. It is speculated that peripheral cytokine IL-1 plays a regulatory role by inhibiting placental microbiota and then acting on B lymphocytes and Treg cells. However, the balance ratio IFN-γ/IL-4 of peripheral cytokines has little effect on the placental microbiota.

In conclusion, normal term maternal systemic immunity interacts with placental flora, especially the negative regulation of peripheral B lymphocytes on placental microbiota and the positive regulation of peripheral Treg cells on placental microbiota. At the same time, the corresponding peripheral cytokine IL-1 and placental microbiota inhibit each other.

## Conclusions

The placental microbiota may be an important factor mediating local immune regulation in the placenta, and placental microbiota participates in regulating the maternal immune system.

### Supplementary Information

**Supplementary Material 1.**

**Supplementary Material 2.**

## Data Availability

The datasets generated and/or analyzed during the current study are not publicly available but are available from the corresponding author on reasonable request.
