# Supplementary material for: The influence of placenta microbiota of normal term pregnant women on immune regulation during pregnancy
Source: BMC Pregnancy Childbirth. 2024 Feb 29;24:171. doi: 10.1186/s12884-024-06353-x (PMC10905846; doi:10.1186/s12884-024-06353-x)
Supplement: Supplementary file 1 — Supplementary Material 1. [file 12884_2024_6353_MOESM1_ESM.docx]

Appendix 1: Correlation coefficients between the top 100 bacterial genera of normal-term placental microbiota and immune cells in placenta tissue

| Placental immune cells | Placental microbiota | R Value |
| --- | --- | --- |
| CD3+CD4+T | Capnocytophaga | -0.427723611 |
| CD3+CD8+T | Acinetobacter | 0.612821261 |
| CD3-CD19+/CD3- | Acinetobacter | -0.501859062 |
| CD3-CD19+/CD3- | Prevotellaceae_NK3B31_group | -0.642930021 |
| CD3-CD56+/CD3- | Capnocytophaga | -0.417119516 |
| CD3-CD56+/CD3- | Leptotrichia | -0.449293721 |
| CD3-CD68+/CD3- | Acidibacter | 0.743612843 |
| CD3-CD68+/CD3- | Acidothermus | 0.672226069 |
| CD3-CD68+/CD3- | Bacteroides | -0.597498322 |
| CD3-CD68+/CD3- | Barnesiella | -0.641189507 |
| CD3-CD68+/CD3- | Blautia | -0.517647059 |
| CD3-CD68+/CD3- | Butyricicoccus | -0.564938363 |
| CD3-CD68+/CD3- | Catenibacterium | -0.582432101 |
| CD3-CD68+/CD3- | Collinsella | -0.515897518 |
| CD3-CD68+/CD3- | Cutibacterium | 0.697718133 |
| CD3-CD68+/CD3- | Enterococcus | -0.603829815 |
| CD3-CD68+/CD3- | Escherichia.Shigella | -0.506627943 |
| CD3-CD68+/CD3- | Fusicatenibacter | -0.567429537 |
| CD3-CD68+/CD3- | Fusobacterium | -0.518853951 |
| CD3-CD68+/CD3- | Lachnoclostridium | -0.501839724 |
| CD3-CD68+/CD3- | Lachnospiraceae_NK4A136_group | -0.552283368 |
| CD3-CD68+/CD3- | Morganella | -0.703720865 |
| CD3-CD68+/CD3- | Neisseria | 0.602160373 |
| CD3-CD68+/CD3- | NK4A214_group | -0.605003462 |
| CD3-CD68+/CD3- | Parasutterella | -0.534947214 |
| CD3-CD68+/CD3- | Romboutsia | -0.56342428 |
| CD3-CD68+/CD3- | Rothia | 0.612247426 |
| CD3-CD68+/CD3- | Sphingomonas | 0.508922243 |
| CD3-CD68+/CD3- | UCG.002 | -0.50298729 |
| CD3-CD68+/CD3- | X.Eubacterium._hallii_group | -0.523677807 |
| CD3-CD68+/CD3- | X.Ruminococcus._gnavus_group | -0.50816209 |
| CD56 dim CD16+ | Prevotellaceae_NK3B31_group | -0.503582377 |
| CD56 dim CD16+ | Rothia | 0.525060938 |
| CD56 high CD16- | Butyricicoccus | 0.58970272 |
| CD56 high CD16- | Capnocytophaga | -0.557692308 |
| CD56 high CD16- | Catenibacterium | 0.570567785 |
| CD56 high CD16- | Erysipelotrichaceae_UCG.003 | 0.548741037 |
| CD56 high CD16- | Fusobacterium | 0.618237259 |
| CD56 high CD16- | Klebsiella | 0.516654472 |
| CD56 high CD16- | Megasphaera | 0.53337658 |
| CD56 high CD16- | Monoglobus | 0.499632247 |
| CD56 high CD16- | NK4A214_group | 0.519143217 |
| CD56 high CD16- | Phascolarctobacterium | 0.52444502 |
| CD56 high CD16- | Roseburia | 0.532544379 |
| CD56 high CD16- | unidentified_Ruminococcaceae | 0.729780728 |
| CD56dimCD16- | Agathobacter | 0.620846811 |
| CD56dimCD16- | Alistipes | 0.680913965 |
| CD56dimCD16- | Anaerostipes | 0.695144928 |
| CD56dimCD16- | Bacteroides | 0.793814433 |
| CD56dimCD16- | Barnesiella | 0.706289266 |
| CD56dimCD16- | Bifidobacterium | 0.617083947 |
| CD56dimCD16- | Blautia | 0.635761762 |
| CD56dimCD16- | Butyricicoccus | 0.818841843 |
| CD56dimCD16- | Catenibacterium | 0.599804462 |
| CD56dimCD16- | Christensenellaceae_R.7_group | 0.566358046 |
| CD56dimCD16- | Clostridium_sensu_stricto_1 | 0.677966286 |
| CD56dimCD16- | Coprococcus | 0.738954834 |
| CD56dimCD16- | Dialister | 0.543783509 |
| CD56dimCD16- | Dorea | 0.710382844 |
| CD56dimCD16- | Enterococcus | 0.703021562 |
| CD56dimCD16- | Erysipelotrichaceae_UCG.003 | 0.575547531 |
| CD56dimCD16- | Escherichia.Shigella | 0.736182956 |
| CD56dimCD16- | Faecalibacterium | 0.792925786 |
| CD56dimCD16- | Fusicatenibacter | 0.682891598 |
| CD56dimCD16- | Fusobacterium | 0.719681643 |
| CD56dimCD16- | Klebsiella | 0.709013434 |
| CD56dimCD16- | Lachnoclostridium | 0.633284242 |
| CD56dimCD16- | Lachnospira | 0.63179538 |
| CD56dimCD16- | Lachnospiraceae_NK4A136_group | 0.617538856 |
| CD56dimCD16- | Lactobacillus | 0.578792342 |
| CD56dimCD16- | Megasphaera | 0.71383162 |
| CD56dimCD16- | Monoglobus | 0.619270133 |
| CD56dimCD16- | Morganella | 0.627212751 |
| CD56dimCD16- | NK4A214_group | 0.680195575 |
| CD56dimCD16- | Parabacteroides | 0.64575804 |
| CD56dimCD16- | Parasutterella | 0.758399738 |
| CD56dimCD16- | Phascolarctobacterium | 0.810057268 |
| CD56dimCD16- | Porphyromonas | 0.697826105 |
| CD56dimCD16- | Prevotella | 0.628704416 |
| CD56dimCD16- | Prevotella_9 | 0.796163012 |
| CD56dimCD16- | Romboutsia | 0.758673446 |
| CD56dimCD16- | Roseburia | 0.531366616 |
| CD56dimCD16- | Ruminococcus | 0.657062875 |
| CD56dimCD16- | Sellimonas | 0.536590297 |
| CD56dimCD16- | Streptococcus | 0.745213549 |
| CD56dimCD16- | Subdoligranulum | 0.685335485 |
| CD56dimCD16- | Sutterella | 0.852150101 |
| CD56dimCD16- | Turicibacter | 0.788469303 |
| CD56dimCD16- | Tyzzerella | 0.524869364 |
| CD56dimCD16- | UBA1819 | 0.556293488 |
| CD56dimCD16- | UCG.002 | 0.636249872 |
| CD56dimCD16- | Veillonella | 0.877967226 |
| CD56dimCD16- | X.Eubacterium._eligens_group | 0.710491397 |
| CD56dimCD16- | X.Eubacterium._hallii_group | 0.703192267 |
| CD56dimCD16- | X.Ruminococcus._gnavus_group | 0.661245257 |
| CD56dimCD16- | X.Ruminococcus._torques_group | 0.616801936 |
| CD56hightCD16+ | Megamonas | -0.518028102 |
| Treg(CD3+CD4+CD25+FOXP3) | Rikenellaceae_RC9_gut_group | 0.54986213 |

Appendix 2: Correlation coefficient between the top 100 bacterial genera of normal-term placental microbiota and immune cells balance in placental tissue

| The balance of placental immune cells | Placental microbiota | R Value |
| --- | --- | --- |
| CD4+/CD8+ | Anaerovibrio | 0.507543371 |
| CD4+/CD8+ | Candidatus_Arthromitus | -0.43673277 |
| CD4+/CD8+ | Capnocytophaga | -0.427723611 |
| CD4+/CD8+ | Catenibacterium | 0.463224791 |
| CD4+/CD8+ | Vibrio | -0.455367304 |
| CD56+CD16+/CD56+CD16- | Acetobacter | -0.677023104 |
| CD56+CD16+/CD56+CD16- | Acidibacter | 0.520836268 |
| CD56+CD16+/CD56+CD16- | Acidothermus | 0.513879483 |
| CD56+CD16+/CD56+CD16- | Agathobacter | -0.725265614 |
| CD56+CD16+/CD56+CD16- | Alistipes | -0.810015606 |
| CD56+CD16+/CD56+CD16- | Alloprevotella | -0.619471722 |
| CD56+CD16+/CD56+CD16- | Anaerostipes | -0.763350047 |
| CD56+CD16+/CD56+CD16- | Anaerovibrio | -0.557455202 |
| CD56+CD16+/CD56+CD16- | Bacteroides | -0.769683799 |
| CD56+CD16+/CD56+CD16- | Barnesiella | -0.64887763 |
| CD56+CD16+/CD56+CD16- | Bifidobacterium | -0.675496872 |
| CD56+CD16+/CD56+CD16- | Blautia | -0.752941176 |
| CD56+CD16+/CD56+CD16- | Butyricicoccus | -0.862759131 |
| CD56+CD16+/CD56+CD16- | Catenibacterium | -0.846558287 |
| CD56+CD16+/CD56+CD16- | Christensenellaceae_R.7_group | -0.762983896 |
| CD56+CD16+/CD56+CD16- | Clostridium_sensu_stricto_1 | -0.721650267 |
| CD56+CD16+/CD56+CD16- | Collinsella | -0.711022081 |
| CD56+CD16+/CD56+CD16- | Coprococcus | -0.847407545 |
| CD56+CD16+/CD56+CD16- | Dialister | -0.815445939 |
| CD56+CD16+/CD56+CD16- | Dorea | -0.725404697 |
| CD56+CD16+/CD56+CD16- | Enterococcus | -0.752578136 |
| CD56+CD16+/CD56+CD16- | Erysipelotrichaceae_UCG.003 | -0.735553555 |
| CD56+CD16+/CD56+CD16- | Escherichia.Shigella | -0.767305692 |
| CD56+CD16+/CD56+CD16- | Faecalibacterium | -0.832106941 |
| CD56+CD16+/CD56+CD16- | Fusicatenibacter | -0.761976807 |
| CD56+CD16+/CD56+CD16- | Fusobacterium | -0.752412139 |
| CD56+CD16+/CD56+CD16- | Haemophilus | -0.623402661 |
| CD56+CD16+/CD56+CD16- | Holdemanella | -0.596880593 |
| CD56+CD16+/CD56+CD16- | Klebsiella | -0.73653638 |
| CD56+CD16+/CD56+CD16- | Lachnoclostridium | -0.754967092 |
| CD56+CD16+/CD56+CD16- | Lachnospira | -0.817539813 |
| CD56+CD16+/CD56+CD16- | Lachnospiraceae_NK4A136_group | -0.600884304 |
| CD56+CD16+/CD56+CD16- | Lactobacillus | -0.709345299 |
| CD56+CD16+/CD56+CD16- | Megamonas | -0.706401957 |
| CD56+CD16+/CD56+CD16- | Megasphaera | -0.761856211 |
| CD56+CD16+/CD56+CD16- | Monoglobus | -0.771297648 |
| CD56+CD16+/CD56+CD16- | Morganella | -0.651884283 |
| CD56+CD16+/CD56+CD16- | NK4A214_group | -0.772313061 |
| CD56+CD16+/CD56+CD16- | Parabacteroides | -0.715342345 |
| CD56+CD16+/CD56+CD16- | Parasutterella | -0.705833129 |
| CD56+CD16+/CD56+CD16- | Phascolarctobacterium | -0.799121583 |
| CD56+CD16+/CD56+CD16- | Porphyromonas | -0.618125136 |
| CD56+CD16+/CD56+CD16- | Prevotella | -0.733195326 |
| CD56+CD16+/CD56+CD16- | Prevotella_9 | -0.840023046 |
| CD56+CD16+/CD56+CD16- | Romboutsia | -0.784664181 |
| CD56+CD16+/CD56+CD16- | Roseburia | -0.710917547 |
| CD56+CD16+/CD56+CD16- | Rothia | 0.514775199 |
| CD56+CD16+/CD56+CD16- | Ruminococcus | -0.843435164 |
| CD56+CD16+/CD56+CD16- | Sellimonas | -0.801756198 |
| CD56+CD16+/CD56+CD16- | Staphylococcus | -0.52430101 |
| CD56+CD16+/CD56+CD16- | Streptococcus | -0.676968542 |
| CD56+CD16+/CD56+CD16- | Subdoligranulum | -0.749632624 |
| CD56+CD16+/CD56+CD16- | Sutterella | -0.843212059 |
| CD56+CD16+/CD56+CD16- | Turicibacter | -0.752412139 |
| CD56+CD16+/CD56+CD16- | Tyzzerella | -0.571474566 |
| CD56+CD16+/CD56+CD16- | UCG.002 | -0.715161869 |
| CD56+CD16+/CD56+CD16- | unidentified_Ruminococcaceae | -0.769234329 |
| CD56+CD16+/CD56+CD16- | Veillonella | -0.725804245 |
| CD56+CD16+/CD56+CD16- | X.Eubacterium._eligens_group | -0.74038542 |
| CD56+CD16+/CD56+CD16- | X.Eubacterium._hallii_group | -0.795871921 |
| CD56+CD16+/CD56+CD16- | X.Ruminococcus._gnavus_group | -0.739279542 |
| CD56+CD16+/CD56+CD16- | X.Ruminococcus._torques_group | -0.762887425 |

Appendix3: Correlation coefficient between the top 100 bacterial genera of normal-term placental microbiota and Placental cytokines

| Placental cytokines | Placental microbiota | R Value |
| --- | --- | --- |
| GM-CSF | Gemella | -0.468250819 |
| GM-CSF | Neisseria | -0.464002527 |
| IL-1 | Acidibacter | 0.501330615 |
| IL-1 | Acidothermus | 0.495533796 |
| IL-1 | Acinetobacter | -0.581683721 |
| IL-1 | Anaerostipes | -0.496318287 |
| IL-1 | Bacteroides | -0.445072884 |
| IL-1 | Butyricicoccus | -0.431186236 |
| IL-1 | Campylobacter | -0.447499285 |
| IL-1 | Clostridium_sensu_stricto_1 | -0.468641293 |
| IL-1 | Coprococcus | -0.422628538 |
| IL-1 | Klebsiella | -0.500544841 |
| IL-1 | Lachnospiraceae_NK4A136_group | -0.536670307 |
| IL-1 | Morganella | -0.453950107 |
| IL-1 | Parasutterella | -0.441120033 |
| IL-1 | Prevotella_9 | -0.429039413 |
| IL-1 | Pseudomonas | -0.460635102 |
| IL-1 | Rikenellaceae_RC9_gut_group | -0.428222476 |
| IL-1 | Romboutsia | -0.485086104 |
| IL-1 | UBA1819 | -0.442171136 |
| IL-1 | UCG.002 | -0.42121541 |
| IL-1 | Veillonella | -0.451411716 |
| IL-1 | Vibrio | -0.461705831 |
| IL-1 | X.Eubacterium._hallii_group | -0.424864909 |
| IL-12 | Gemella | -0.406680846 |
| IL-12 | Neisseria | -0.420805248 |
| IL-13 | Actinomyces | -0.412261576 |
| IL-13 | Cutibacterium | -0.565536196 |
| IL-13 | Enterococcus | 0.438207178 |
| IL-13 | Rothia | -0.690865642 |
| IL-18 | Acidothermus | 0.442786999 |
| IL-18 | Bacteroides | -0.424532405 |
| IL-18 | Lachnospiraceae_NK4A136_group | -0.458007006 |
| IL-18 | Parasutterella | -0.503526256 |
| IL-18 | Porphyromonas | -0.474034736 |
| IL-18 | Prevotella_9 | -0.453139699 |
| IL-18 | Prevotellaceae_NK3B31_group | -0.4546725 |
| IL-18 | Rikenellaceae_RC9_gut_group | -0.469675875 |
| IL-18 | Veillonella | -0.416597104 |
| IL-18 | X.Eubacterium._eligens_group | -0.411910462 |
| IL-2 | Acinetobacter | -0.426365031 |
| IL-5 | Acidibacter | -0.653808377 |
| IL-5 | Acidothermus | -0.553855195 |
| IL-5 | Corynebacterium | -0.459610321 |
| IL-5 | Holdemanella | 0.437304229 |
| IL-5 | Rikenellaceae_RC9_gut_group | 0.426278593 |
| IL-5 | Rothia | -0.705030292 |
| IL-5 | Sellimonas | 0.409472541 |
| IL-6 | Campylobacter | 0.444797364 |
| IL-6 | Cutibacterium | -0.440712213 |
| IL-6 | Lactobacillus | 0.404699892 |
| TNF-a | Acidibacter | 0.611228049 |
| TNF-a | Acidothermus | 0.622378928 |
| TNF-a | Acinetobacter | -0.658112223 |
| TNF-a | Agathobacter | -0.406843232 |
| TNF-a | Alloprevotella | -0.443670498 |
| TNF-a | Anaerostipes | -0.522804902 |
| TNF-a | Bacteroides | -0.460635059 |
| TNF-a | Barnesiella | -0.453205983 |
| TNF-a | Butyricicoccus | -0.4379212 |
| TNF-a | Clostridium_sensu_stricto_1 | -0.519921928 |
| TNF-a | Coprococcus | -0.467294031 |
| TNF-a | Escherichia.Shigella | -0.458124875 |
| TNF-a | Klebsiella | -0.567073655 |
| TNF-a | Lachnospiraceae_NK4A136_group | -0.60879031 |
| TNF-a | Morganella | -0.487252663 |
| TNF-a | NK4A214_group | -0.413264702 |
| TNF-a | Parabacteroides | -0.454982778 |
| TNF-a | Parasutterella | -0.493876798 |
| TNF-a | Prevotella_9 | -0.488449286 |
| TNF-a | Prevotellaceae_NK3B31_group | -0.44766316 |
| TNF-a | Pseudomonas | -0.405740388 |
| TNF-a | Rikenellaceae_RC9_gut_group | -0.545659389 |
| TNF-a | Romboutsia | -0.509795579 |
| TNF-a | Ruminococcus | -0.438058174 |
| TNF-a | Staphylococcus | -0.451163813 |
| TNF-a | UBA1819 | -0.419423185 |
| TNF-a | UCG.002 | -0.511676317 |
| TNF-a | Veillonella | -0.413321969 |
| TNF-a | X.Eubacterium._eligens_group | -0.454264601 |
| TNF-a | X.Eubacterium._hallii_group | -0.454428071 |

Appendix4: Correlation coefficient between the top 100 bacterial genera of normal-term placental microbiota and IFN-r、IL-4及IFN-r/IL-4 in placental tissue

| Placental cytokines | Placental microbiota | R Value |
| --- | --- | --- |
| IFN/IL4 | Acidibacter | -0.412738089 |
| IFN/IL4 | Acidothermus | -0.409079846 |
| IFN/IL4 | Escherichia.Shigella | 0.493692957 |
| IFN/IL4 | Klebsiella | 0.444590109 |
| IFN/IL4 | Morganella | 0.502264296 |
| IFN/IL4 | UCG.002 | 0.45288234 |
| IFN-γ | Actinomyces | -0.47920882 |
| IFN-γ | Anaerostipes | -0.412353078 |
| IFN-γ | Gemella | -0.420997149 |
| IFN-γ | Haemophilus | -0.434908185 |
| IFN-γ | Parasutterella | -0.523500091 |
| IFN-γ | Porphyromonas | -0.547108774 |
| IFN-γ | UBA1819 | -0.426126806 |
| IFN-γ | Veillonella | -0.427449553 |
| IL-4 | Acetobacter | -0.466072756 |
| IL-4 | Acidibacter | 0.488108216 |
| IL-4 | Acidothermus | 0.560008332 |
| IL-4 | Acinetobacter | -0.475424097 |
| IL-4 | Agathobacter | -0.474614118 |
| IL-4 | Anaerostipes | -0.444794167 |
| IL-4 | Bacteroides | -0.517616355 |
| IL-4 | Barnesiella | -0.408221093 |
| IL-4 | Bilophila | -0.442961132 |
| IL-4 | Butyricicoccus | -0.51568085 |
| IL-4 | Clostridium_sensu_stricto_1 | -0.524711828 |
| IL-4 | Coprococcus | -0.47039603 |
| IL-4 | Dorea | -0.406602488 |
| IL-4 | Enterococcus | -0.429503957 |
| IL-4 | Escherichia.Shigella | -0.608222768 |
| IL-4 | Fusicatenibacter | -0.417338756 |
| IL-4 | Fusobacterium | -0.511806614 |
| IL-4 | Klebsiella | -0.5228663 |
| IL-4 | Lachnospiraceae_NK4A136_group | -0.41840735 |
| IL-4 | Monoglobus | -0.493556468 |
| IL-4 | Morganella | -0.641620806 |
| IL-4 | NK4A214_group | -0.485022681 |
| IL-4 | Parabacteroides | -0.54807221 |
| IL-4 | Paraprevotella | -0.442205669 |
| IL-4 | Parasutterella | -0.539711723 |
| IL-4 | Phascolarctobacterium | -0.474587333 |
| IL-4 | Prevotella_9 | -0.460114432 |
| IL-4 | Prevotellaceae_NK3B31_group | -0.442289333 |
| IL-4 | Romboutsia | -0.470396347 |
| IL-4 | Roseburia | -0.483759839 |
| IL-4 | Ruminococcus | -0.442412629 |
| IL-4 | Streptococcus | -0.482053525 |
| IL-4 | Turicibacter | -0.408592728 |
| IL-4 | UCG.002 | -0.564698639 |
| IL-4 | Veillonella | -0.552406013 |
| IL-4 | X.Eubacterium._eligens_group | -0.558504063 |
| IL-4 | X.Eubacterium._hallii_group | -0.457480851 |
| IL-4 | X.Ruminococcus._gnavus_group | -0.47595758 |

Appendix 5: Correlation coefficient between the top 100 bacterial genera of normal-term placental microbiota and peripheral immune cells

| Peripheral blood immune cells | Placental microbiota | R Value |
| --- | --- | --- |
| CD3+CD8+T | Alloprevotella | 0.4732678 |
| CD3+CD8+T | Enterococcus | 0.435839527 |
| CD3+CD8+T | Lachnospiraceae_NK4A136_group | 0.433455796 |
| CD3+CD8+T | Rikenellaceae_RC9_gut_group | 0.671912849 |
| CD3+CD8+T | UBA1819 | 0.449623098 |
| CD3-CD19+/CD3- | Agathobacter | -0.48638035 |
| CD3-CD19+/CD3- | Alistipes | -0.469554973 |
| CD3-CD19+/CD3- | Anaerostipes | -0.429224068 |
| CD3-CD19+/CD3- | Bacteroides | -0.555116164 |
| CD3-CD19+/CD3- | Barnesiella | -0.471296029 |
| CD3-CD19+/CD3- | Butyricicoccus | -0.452289848 |
| CD3-CD19+/CD3- | Clostridium_sensu_stricto_1 | -0.467111828 |
| CD3-CD19+/CD3- | Coprococcus | -0.50440414 |
| CD3-CD19+/CD3- | Dorea | -0.48435769 |
| CD3-CD19+/CD3- | Escherichia.Shigella | -0.418541422 |
| CD3-CD19+/CD3- | Faecalibacterium | -0.490482092 |
| CD3-CD19+/CD3- | Fusobacterium | -0.43554977 |
| CD3-CD19+/CD3- | Holdemanella | -0.433995185 |
| CD3-CD19+/CD3- | Morganella | -0.476918792 |
| CD3-CD19+/CD3- | Parabacteroides | -0.448737879 |
| CD3-CD19+/CD3- | Paraprevotella | -0.531487028 |
| CD3-CD19+/CD3- | Parasutterella | -0.554818911 |
| CD3-CD19+/CD3- | Porphyromonas | -0.451747012 |
| CD3-CD19+/CD3- | Prevotella_9 | -0.430625702 |
| CD3-CD19+/CD3- | Prevotellaceae_NK3B31_group | -0.430035588 |
| CD3-CD19+/CD3- | Ralstonia | 0.600137935 |
| CD3-CD19+/CD3- | Rikenellaceae_RC9_gut_group | -0.418713023 |
| CD3-CD19+/CD3- | Romboutsia | -0.467095726 |
| CD3-CD19+/CD3- | Sellimonas | -0.465713966 |
| CD3-CD19+/CD3- | Subdoligranulum | -0.512249835 |
| CD3-CD19+/CD3- | Treponema | -0.430423721 |
| CD3-CD19+/CD3- | UBA1819 | -0.471548759 |
| CD3-CD19+/CD3- | UCG.002 | -0.463809687 |
| CD3-CD19+/CD3- | Veillonella | -0.422411388 |
| CD3-CD19+/CD3- | X.Eubacterium._eligens_group | -0.441800285 |
| CD3-CD19+/CD3- | X.Eubacterium._hallii_group | -0.502354481 |
| CD3-CD56+/CD3- | Acidaminococcus | -0.644193279 |
| CD3-CD56+/CD3- | Capnocytophaga | 0.433289334 |
| CD3-CD56+/CD3- | Leptotrichia | -0.406093504 |
| CD3-CD68+/CD3- | Campylobacter | -0.647108408 |
| CD3-CD68+/CD3- | Veillonella | -0.510360055 |
| CD56 dim CD16+ | Haemophilus | 0.556200051 |
| CD56 dim CD16+ | Prevotella_7 | 0.517923804 |
| CD56 dim CD16+ | Rikenellaceae_RC9_gut_group | 0.653393436 |
| CD56 dim CD16+ | UBA1819 | 0.511443129 |
| CD56 high CD16- | Campylobacter | 0.613940614 |
| CD56 high CD16- | Pseudomonas | 0.745392529 |
| CD56+CD16+/CD56+CD16- | Pseudomonas | -0.51508476 |
| CD56dimCD16- | Bilophila | 0.520257119 |
| CD56dimCD16- | Pseudomonas | 0.686304132 |
| CD56dimCD16- | Vibrio | 0.574060583 |
| Treg(CD3+CD4+CD25+FOXP3) | Acetobacter | 0.586879546 |
| Treg(CD3+CD4+CD25+FOXP3) | Acidibacter | -0.53169413 |
| Treg(CD3+CD4+CD25+FOXP3) | Acidothermus | -0.510185057 |
| Treg(CD3+CD4+CD25+FOXP3) | Acinetobacter | 0.664714766 |
| Treg(CD3+CD4+CD25+FOXP3) | Agathobacter | 0.594640817 |
| Treg(CD3+CD4+CD25+FOXP3) | Akkermansia | 0.538864578 |
| Treg(CD3+CD4+CD25+FOXP3) | Alistipes | 0.538647853 |
| Treg(CD3+CD4+CD25+FOXP3) | Alloprevotella | 0.694952788 |
| Treg(CD3+CD4+CD25+FOXP3) | Bacteroides | 0.49890305 |
| Treg(CD3+CD4+CD25+FOXP3) | Barnesiella | 0.555391979 |
| Treg(CD3+CD4+CD25+FOXP3) | Bilophila | 0.531507467 |
| Treg(CD3+CD4+CD25+FOXP3) | Blautia | 0.531178318 |
| Treg(CD3+CD4+CD25+FOXP3) | Collinsella | 0.571219481 |
| Treg(CD3+CD4+CD25+FOXP3) | Cutibacterium | -0.627148282 |
| Treg(CD3+CD4+CD25+FOXP3) | Dialister | 0.506357658 |
| Treg(CD3+CD4+CD25+FOXP3) | Fusicatenibacter | 0.566555435 |
| Treg(CD3+CD4+CD25+FOXP3) | Klebsiella | 0.519739003 |
| Treg(CD3+CD4+CD25+FOXP3) | Lachnoclostridium | 0.526372414 |
| Treg(CD3+CD4+CD25+FOXP3) | Lachnospira | 0.535928144 |
| Treg(CD3+CD4+CD25+FOXP3) | Lachnospiraceae_NK4A136_group | 0.528246377 |
| Treg(CD3+CD4+CD25+FOXP3) | Lactobacillus | 0.538993474 |
| Treg(CD3+CD4+CD25+FOXP3) | Morganella | 0.561037513 |
| Treg(CD3+CD4+CD25+FOXP3) | Paraprevotella | 0.648335608 |
| Treg(CD3+CD4+CD25+FOXP3) | Pseudomonas | 0.547499439 |
| Treg(CD3+CD4+CD25+FOXP3) | Roseburia | 0.52977129 |
| Treg(CD3+CD4+CD25+FOXP3) | Rothia | -0.527061459 |
| Treg(CD3+CD4+CD25+FOXP3) | Ruminococcus | 0.523850243 |
| Treg(CD3+CD4+CD25+FOXP3) | X.Eubacterium._hallii_group | 0.55373381 |

Appendix 6: Correlation coefficient between the top 100 bacterial genera of normal-term placental microbiota and Peripheral blood cytokines

| Peripheral blood cytokines | Placental microbiota | R Value |
| --- | --- | --- |
| IFN-r | Acetobacter | 0.410304357 |
| IFN-r | Erysipelotrichaceae_UCG.003 | 0.41091677 |
| IFN-r/IL-4 | Acidaminococcus | -0.472531565 |
| IFN-r/IL-4 | Pseudomonas | 0.466196123 |
| IL-1 beta | Acidibacter | 0.41893293 |
| IL-1 beta | Anaerovibrio | -0.458068267 |
| IL-1 beta | Bifidobacterium | -0.407340517 |
| IL-1 beta | Catenibacterium | -0.424461771 |
| IL-1 beta | Holdemanella | -0.512212191 |
| IL-1 beta | Prevotellaceae_NK3B31_group | -0.521578036 |
| IL-1 beta | Rikenellaceae_RC9_gut_group | -0.73799658 |
| IL-1 beta | Sellimonas | -0.415152798 |
| IL-1 beta | Staphylococcus | -0.416678879 |
| IL-1 beta | UBA1819 | -0.551373557 |
| IL-13 | Prevotella_7 | -0.614719844 |
| IL-13 | Sphingomonas | -0.546138663 |
| IL-13 | Thauera | 0.405176987 |
| IL-18 | Anaerovibrio | -0.541906499 |
| IL-18 | Catenibacterium | -0.475248457 |
| IL-18 | Holdemanella | -0.459224308 |
| IL-18 | Prevotellaceae_NK3B31_group | -0.533528371 |
| IL-18 | Rikenellaceae_RC9_gut_group | -0.641499063 |
| IL-18 | UBA1819 | -0.443683447 |
| IL-4 | Acidaminococcus | -0.473010239 |
| IL-4 | Pseudomonas | 0.440110017 |
| IL-6 | Pseudomonas | 0.583169783 |
| IL-6 | Rikenellaceae_RC9_gut_group | -0.405037451 |
