# Supplementary material for: The influence of placenta microbiota of normal term pregnant women on immune regulation during pregnancy
Source: BMC Pregnancy Childbirth. 2024 Feb 29;24:171. doi: 10.1186/s12884-024-06353-x (PMC10905846; doi:10.1186/s12884-024-06353-x)
Supplement: Supplementary file 2 — Supplementary Material 2. [file 12884_2024_6353_MOESM2_ESM.docx]

**Illustration**


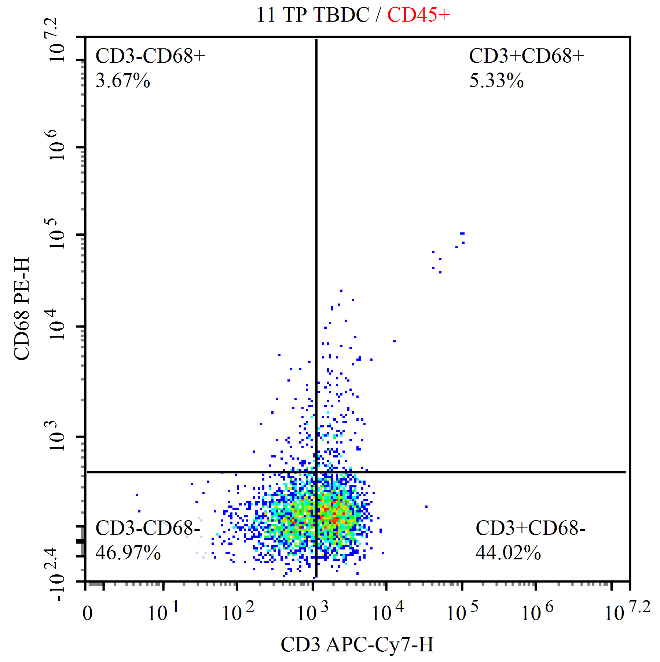

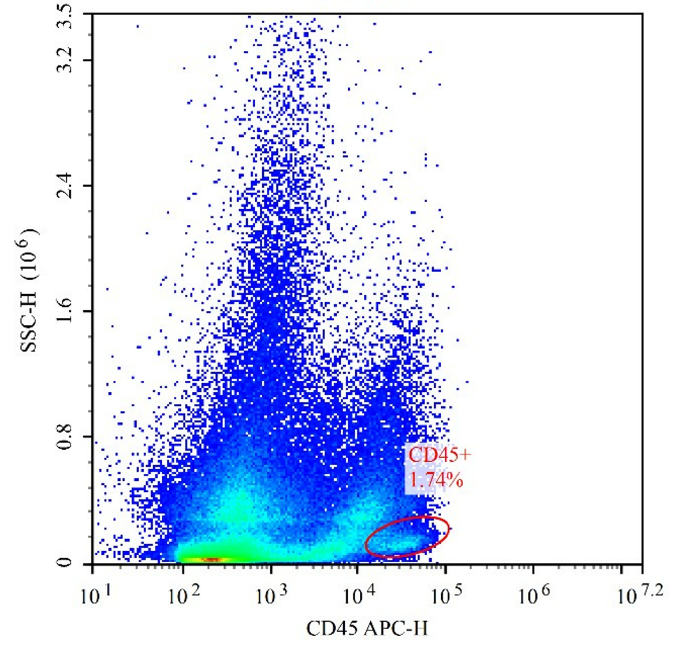


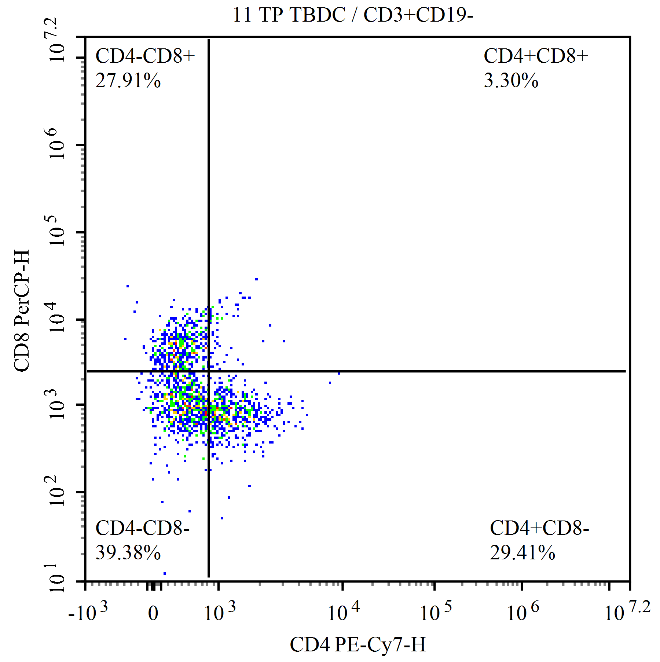

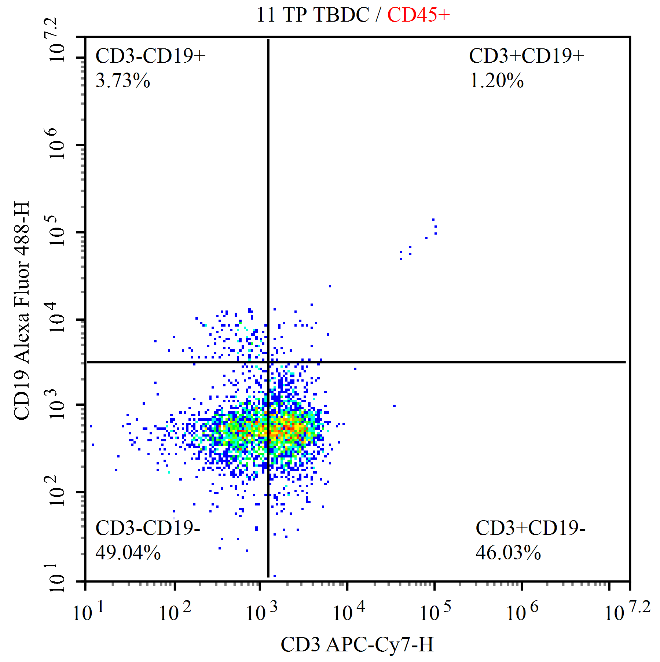


Illustration1. Flow cytometry scatter plot of T lymphocyte subtypes, B lymphocytes, and macrophages in placental tissue


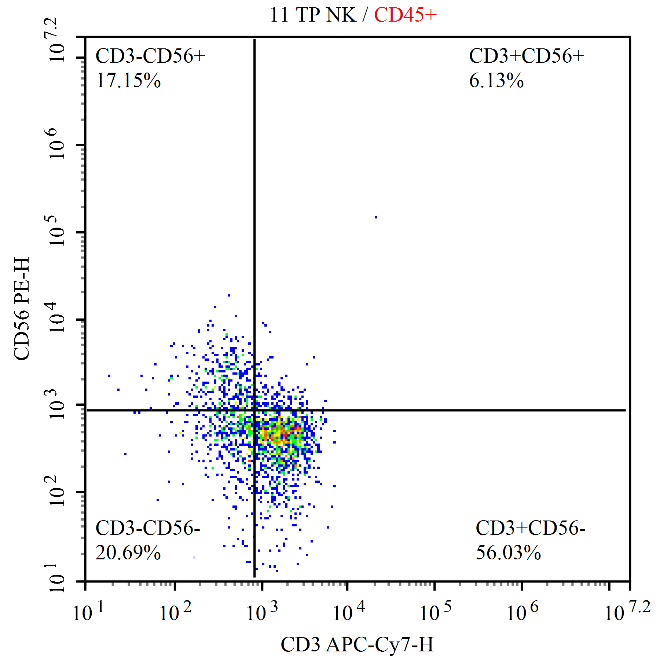

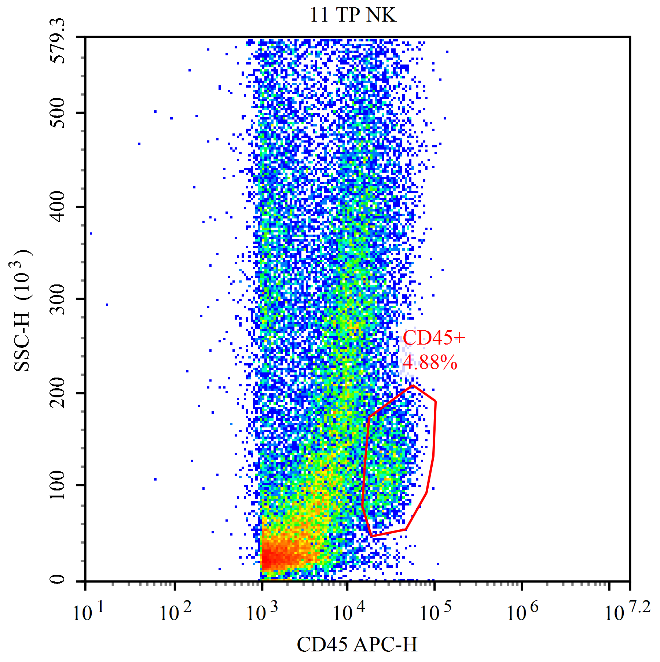


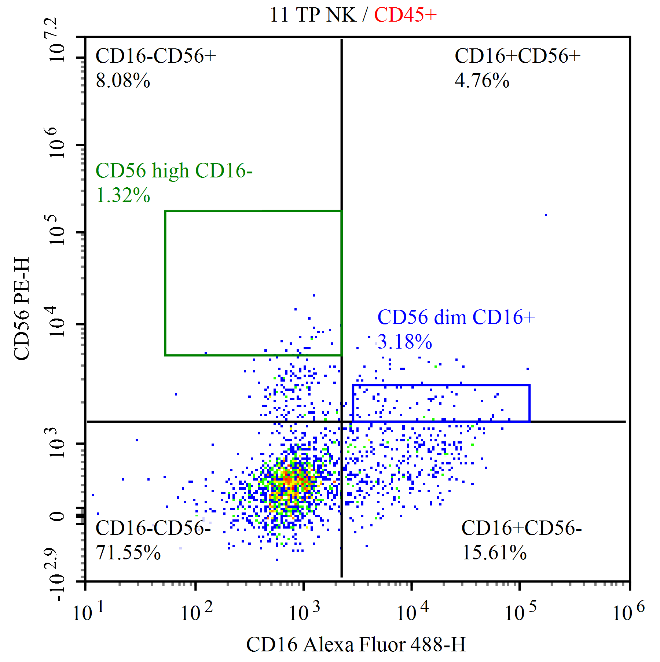


Illustration 2. Flow cytometry scatter plot of NK cells and subtypes in placental tissue


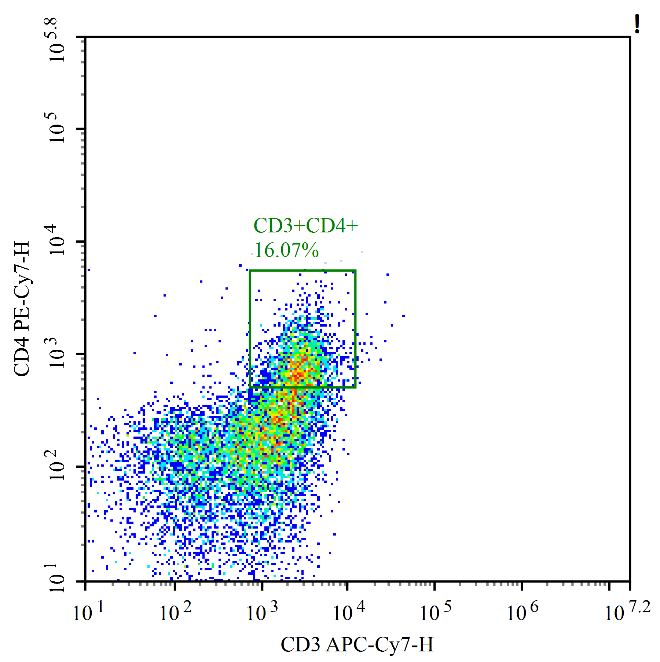

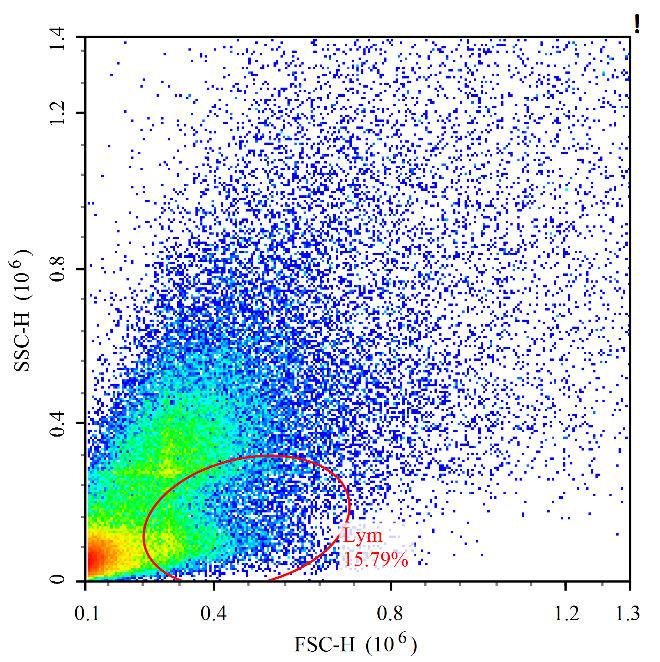


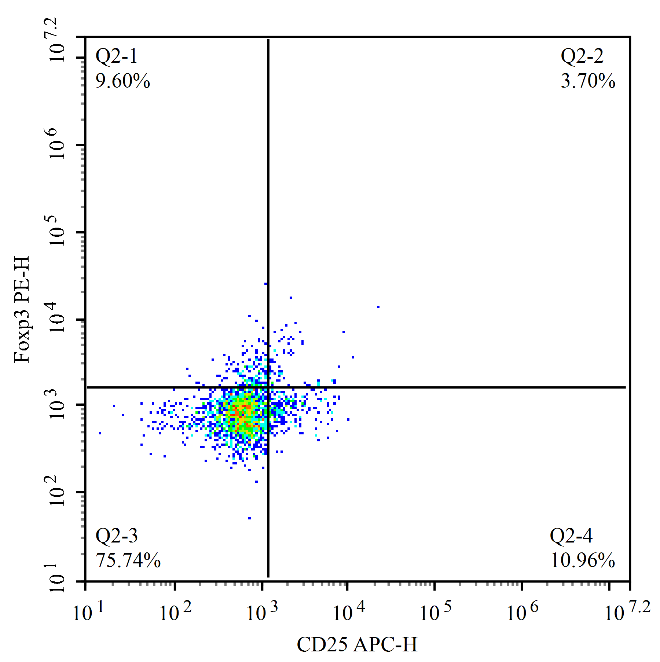


Illustration 3. Flow cytometry scatter plot of Regulating T Cells (Treg Cells) in Placenta tissue


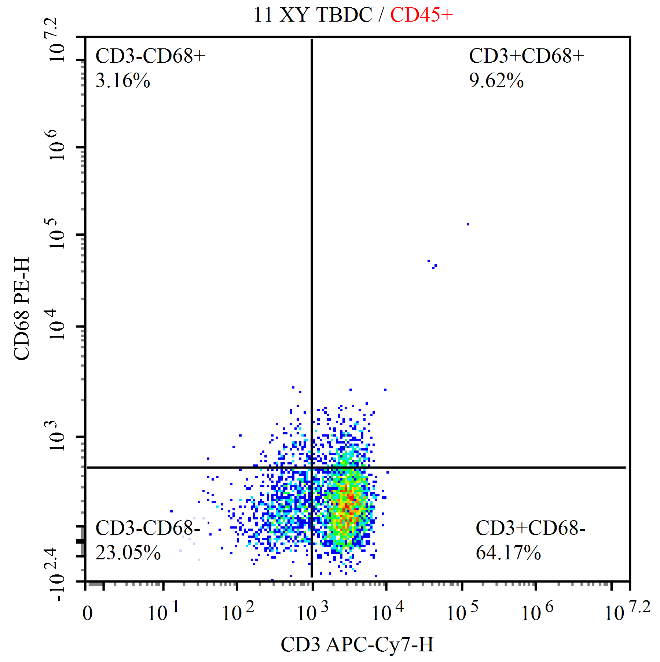

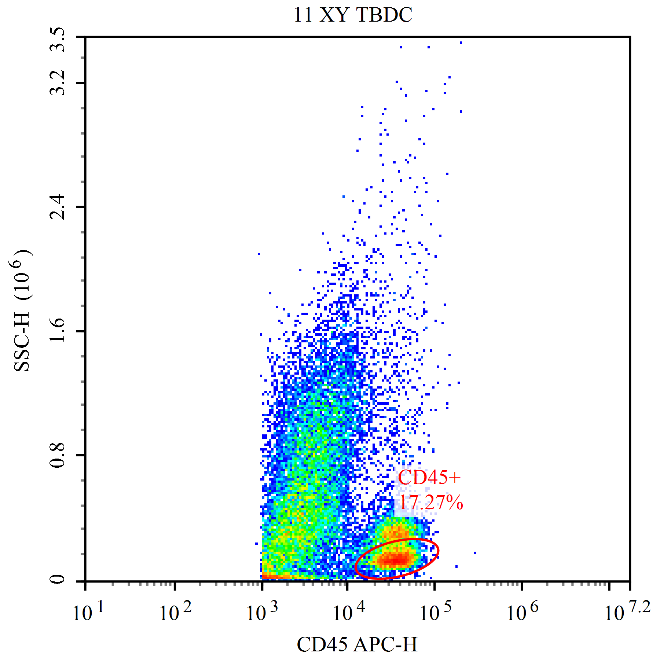


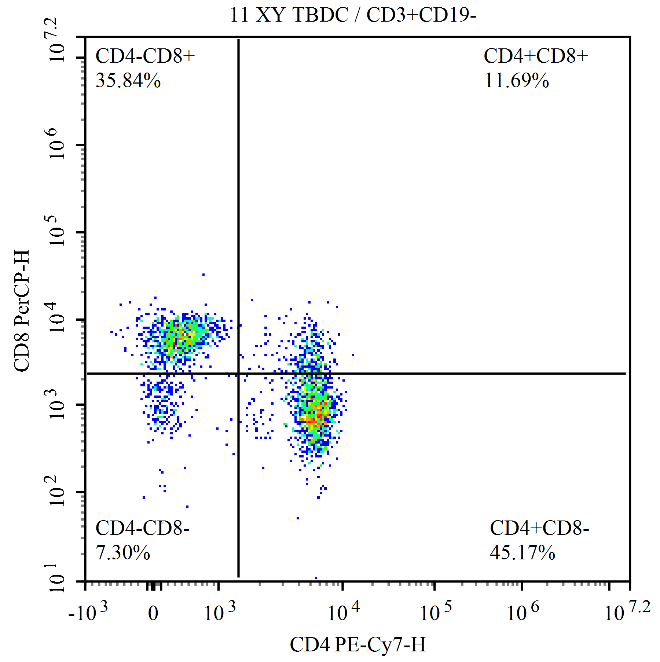

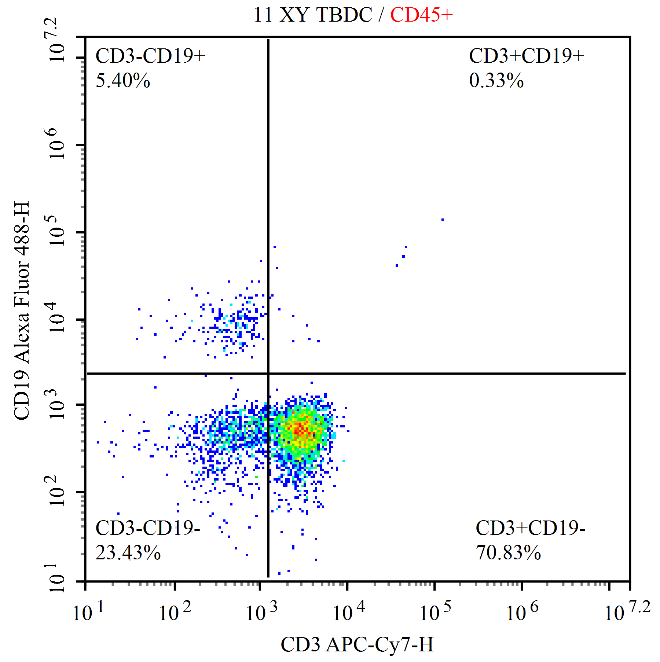


Illustration 4. Flow cytometry scatter plot of T lymphocyte subtypes, B lymphocyte and macrophage populations in peripheral blood


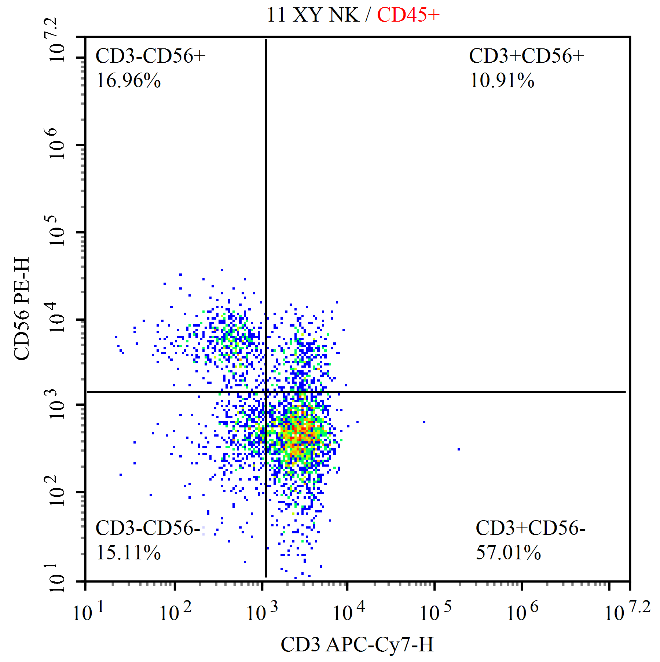


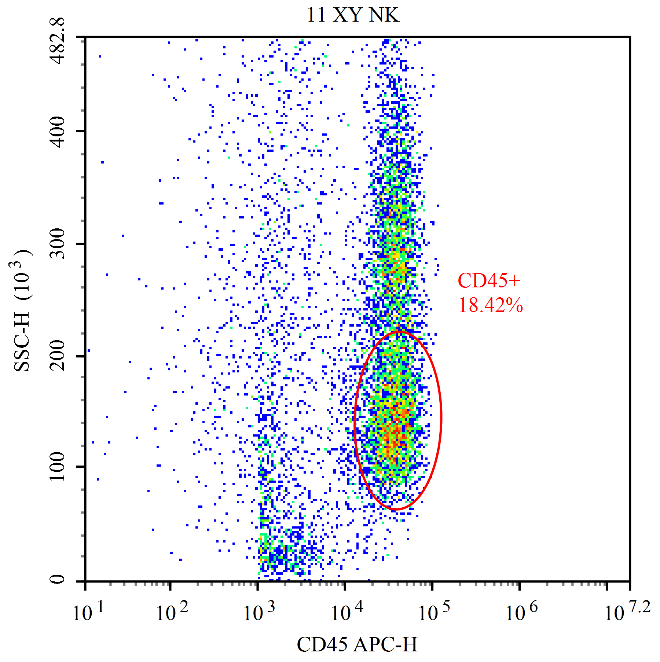


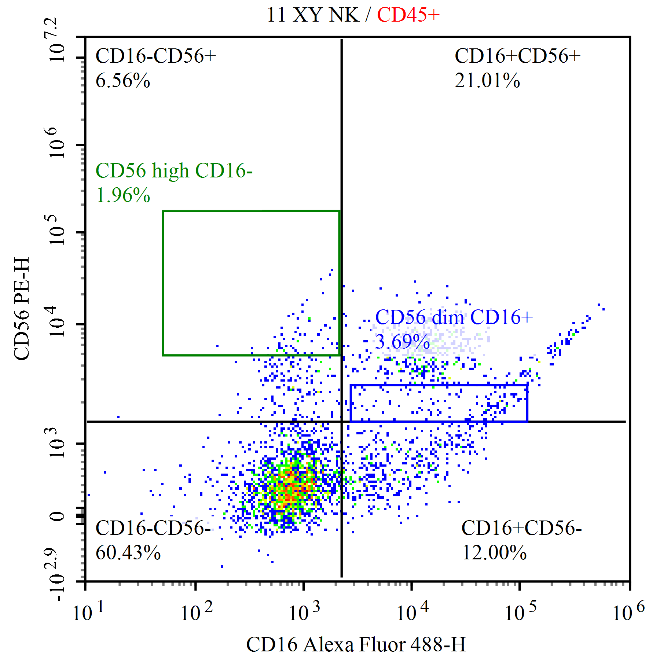


Illustration 5. Flow cytometry scatter plot of NK cells and subtypes in peripheral blood


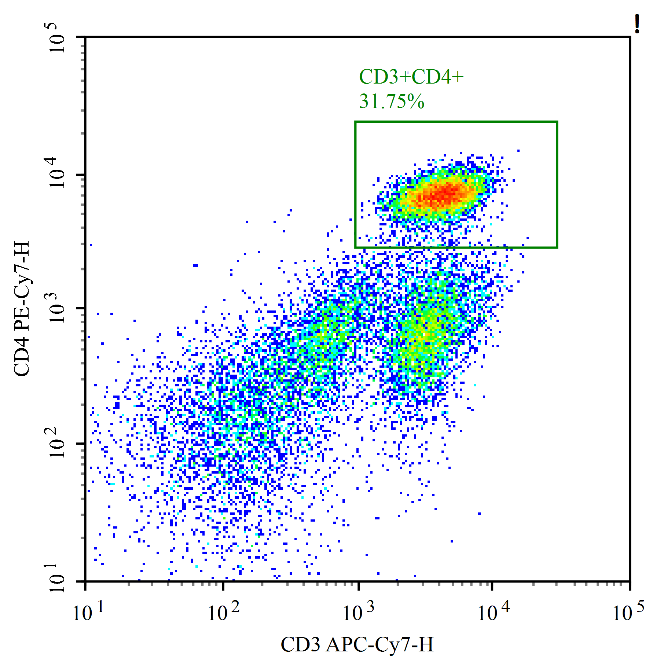

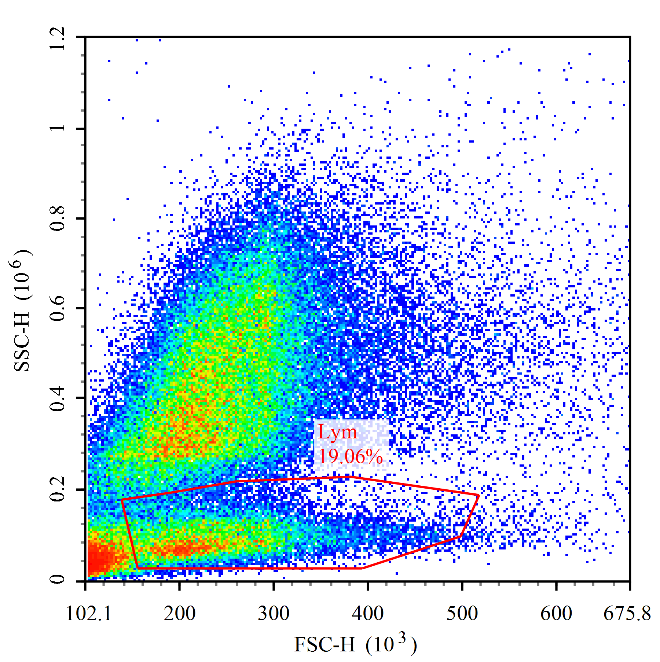


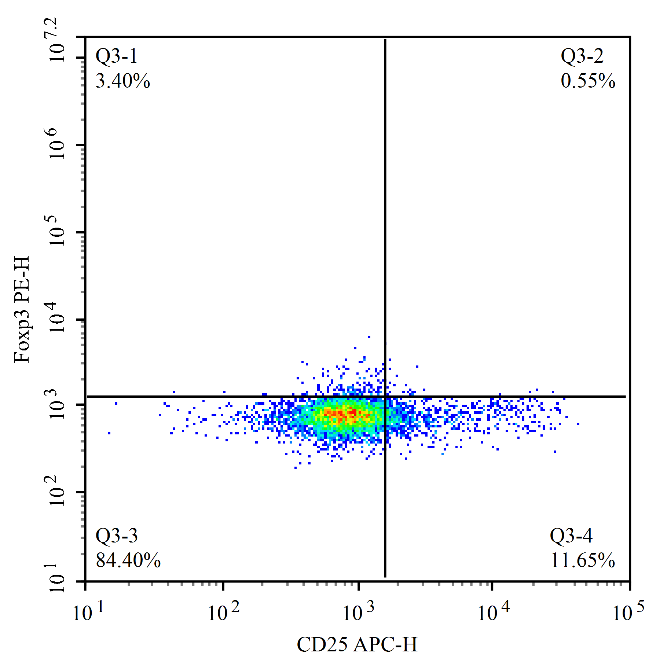


Illustration 6. Flow cytometry scatter plot of Regulating T Cells (Treg Cells) in peripheral blood
